# Sea Cucumbers Metabolites as Potent Anti-Cancer Agents

**DOI:** 10.3390/md13052909

**Published:** 2015-05-12

**Authors:** Naveena B. Janakiram, Altaf Mohammed, Chinthalapally V. Rao

**Affiliations:** Center for Cancer Prevention and Drug Development, Department of Medicine, Hem-Onc Section, University of Oklahoma Health Sciences Center, Oklahoma City, OK 73104, USA; E-Mail: amohamme@ouhsc.edu

**Keywords:** Frondanol A5, sea cucumber, colon cancer, anti-inflammation, aberrant crypt foci

## Abstract

Sea cucumbers and their extracts have gained immense popularity and interest among researchers and nutritionists due to their nutritive value, potential health benefits, and use in the treatment of chronic inflammatory diseases. Many areas of the world use sea cucumbers in traditional foods and folk medicine. Though the actual components and their specific functions still remain to be investigated, most sea cucumber extracts are being studied for their anti-inflammatory functions, immunostimulatory properties, and for cancer prevention and treatment. There is large scope for the discovery of additional bioactive, valuable compounds from this natural source. Sea cucumber extracts contain unique components, such as modified triterpene glycosides, sulfated polysaccharides, glycosphingolipids, and esterified phospholipids. Frondanol A5, an isopropyl alcohol/water extract of the enzymatically hydrolyzed epithelia of the edible North Atlantic sea cucumber, *Cucumaria frondosa*, contains monosulfated triterpenoid glycoside Frondoside A, the disulfated glycoside Frondoside B, the trisulfated glycoside Frondoside C, 12-methyltetradecanoic acid, eicosapentaenoic acid, and fucosylated chondroitin sulfate. We have extensively studied the efficacy of this extract in preventing colon cancer in rodent models. In this review, we discuss the anti-inflammatory, immunostimulatory, and anti-tumor properties of sea cucumber extracts.

## 1. Introduction

At the present time, ~60% of approved cancer treatment drugs are of natural origin [1,2,3]. Natural products that exist in marine animals and plants function as anti-mutagenic and anti-carcinogenic and may inhibit one or more stages of carcinogenesis by preventing or delaying cancer development [4]. An estimated 14,000 pharmacologically active compounds have been isolated from marine plants and animals, suggesting an existence of immense diversity within this environment. The marine environment is therefore a rich source for discovering novel lead compounds for the development of new anti-cancer drugs [5,6] and cancer-preventive nutraceuticals. A comprehensive survey of pharmacologic activity was conducted over a period of 15 years by the U.S. National Cancer Institute and found that 4% of the marine species (mainly animals) examined contained anti-tumor compounds. Potential sources of new types of biologically active compounds isolated from marine echinoderms are being applied in biomedical field.

In recent years, attention has been devoted to developing bioactive agents from natural food sources to produce pharmaceutical grade anti-inflammatory supplements. Sea cucumbers are nutrient-rich, invertebrate deep-sea dwellers that have been used for centuries as an anti-inflammatory and anti-disease food source and for treating ailments in Korea, Japan, Indonesia, and China [7,8,9]. Sea cucumbers are usually soft-bodied echinoderms, looking like a cucumber, and they are a diverse group of flexible, elongated, worm-like organisms, with a leathery skin and gelatinous body. Habitually, they tend to live on the sea floor in deep seas. Sea cucumbers are reported in Chinese and Malaysian literature as they are recognized as a tonic and traditional remedy for various diseases. The export and consumption of bioactive components extracted from marine sea cucumbers has increased in western markets as these components become available in supplements for various diseases [10,11,12,13,14,15]. Hence, a comprehensive approach to utilizing biologically active agents derived from natural foods for wellness and towards disease prevention and treatment is necessary.

**Figure 1:**
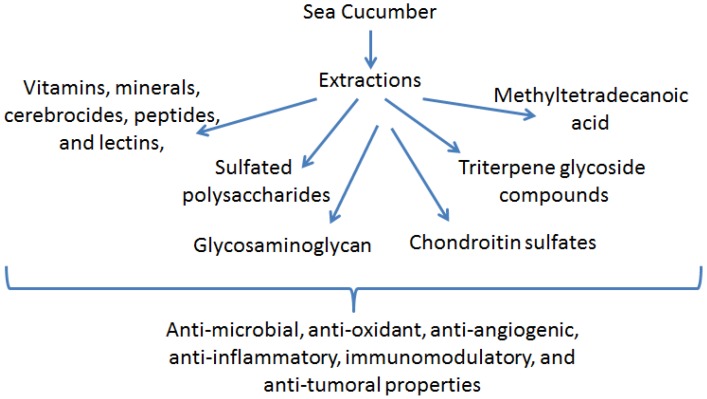
Bioactive components of sea cucumber extracts and their biological effects on various human cancer cells and cancer animal models.

Sea cucumbers consist of vitamins, minerals, cerebrocides, peptides, and lectins, and also contain unique molecules, such as sulfated polysaccharides, 12-methyltetradecanoic acid (12-MTA), philinopside E, triterpene glycoside compounds, glycosaminoglycan, and chondroitin sulfates [16,17,18,19,20,21,22,23,24,25,26,27]. In sufficient quantities, these unique compounds are known to possess anti-microbial, anti-oxidant, anti-angiogenic, anti-inflammatory, immunomodulatory, and anti-tumoral properties [28,29,30,31] (Figure 1). As supplements, these sea cucumber extracts have been shown to suppress inflammation and increase innate immune responses. In this review, we will discuss the anti-inflammatory, anti-tumorigenic, and immune properties of bioactive agents extracted from sea cucumbers.

## 2. Anti-Inflammatory Function of Sea Cucumber Extracts

Inflammation reflects a sequence of events that occurs in response to injury or foreign body entry; this process is highly coordinated and involves various cell types [32]. A normal inflammatory response is characterized by the infiltration of leukocytes and release of other activated inflammatory mediators at the injury/infection site, and eventually will be resolved or regulated with the release of anti-inflammatory mediators [33]. This action is needed to restrict the ongoing inflammation and stop its development into chronic inflammation. Persistent, long-lasting inflammation may lead to inflammatory disease and, eventually, cancer [33,34]. Although several anti-inflammatory agents, like non-steroidal anti-inflammatory drugs (NSAIDs), are available, their use is restricted in dosage or intervals and special precautions are advised due to their gastrointestinal toxicity [35]. Therefore, there is a need for the development of natural anti-inflammatory agents that have the potential to self-limit or resolve inflammatory events, without progressing into chronic inflammation.

Sea cucumber extracts are reported to affect soft tissue repair. Different sea cucumber extracts display different functions, with varied effects when tested *in vitro* or *in vivo*. Compared with the high dose (10 mg/kg), low-dose Stichopus sp1 extract (1 mg/kg) was shown to promote healing properties in rabbits with fractures; these findings indicate that doses are important, and these compounds may not produce dose-dependent effects [36]. The major fatty acids in sea cucumber extracts, EPA and DHA, contribute to tissue/wound healing [37]. EPA and DHA are known *n*3 fatty acids, which inhibit prostaglandin synthesis by suppressing COX-2 and 5-LOX expression under inflammatory conditions, and also act as anti-thrombotics, which accelerate the healing process [38] (Figure 1). These tissue repair properties might play a role during tumor development, when the unique fatty acid content may help to inhibit cancerous transformation of epithelial cells.

Patents by Collins provide wide information on the possible use of bioactive components of sea cucumber tissue fractions as preventive or therapeutic agents to curb inflammation [39,40]. The anti-inflammatory properties of Australian sea cucumbers with a sea plant formulation (95% *w*/*w* *H.* (*Microthele*) *nobilis*, *H.* (*Microthele*) *axiologa* and *Stichopus variegatus*) and sea plant (5% *w*/*w* *Sargassum pallidum*) have been well demonstrated in male Dark Agouti or hooded Wistar rats. Although this formulation produced hypotensive side effects, a dose of 50 mg/kg (ip) was equally effective in inhibiting paw inflammation when compared with 150 mg/kg of aspirin [41]. *Holothuria tubulosa*, *Leptogorgia ceratophyta*, *Coscinasterias tenuispina*, and *Phallusia fumigata* extracts were shown to be effective in down regulating pro-inflammatory marker cyclooxygenase (COX) activity in inflamed mouse tissues [42]. As it is well documented that COX-2 is involved in carcinogenesis, sea cucumber extracts should be further analyzed for their anti-cancer properties.

**Figure 2:**
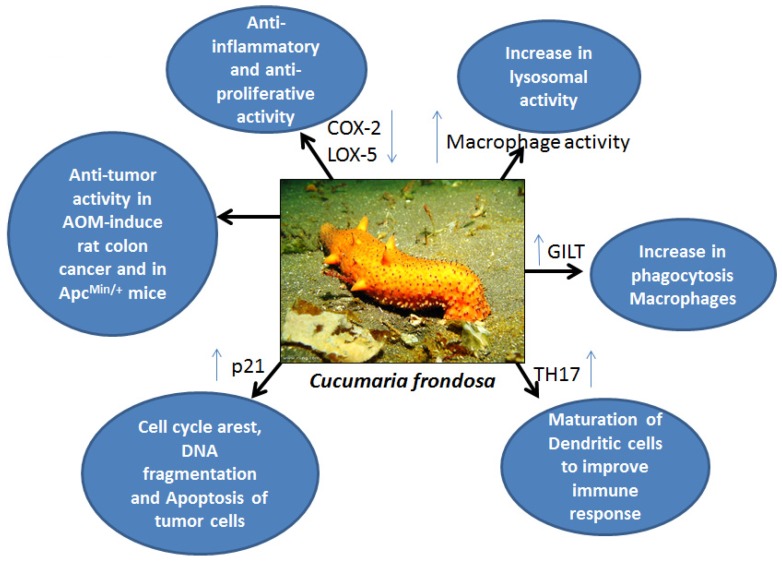
Sea cucumber and its various effects on molecular targets and over all response upon treatment in *in vitro* and *in vivo* cancer models.

**Figure 3:**
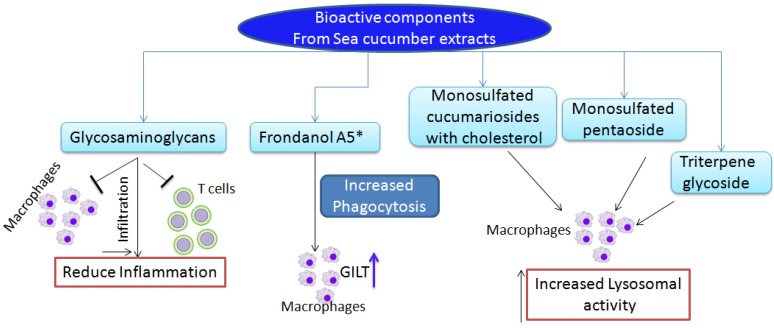
Bioactive compounds isolated from sea cucumbers show immunomodulatory effects and improved immune responses by modulating innate immune cells. * Frondanol A5 consists of monosulfated triterpenoid glycoside Frondoside A, disulfated glycoside Frondoside B, trisulfated glycoside Frondoside C, eicosapentaenoic acid, 12-methyltetradecanoic acid, and fucosylated chondroitin sulfate, as well as canthaxanthin/astaxanthin in small quantities.

Heparin analogues obtained from the sea cucumber *Styela plicata* produced anti-inflammatory effects in a rat model of colon inflammation. Subcutaneous administration of GAGs (4 or 8 mg/kg per day) over seven days reduced colitis induced by 2,4,6-trinitrobenzene sulfonic acid (TNBS) in a Wistar rat. Furthermore, treatment group animals showed less infiltration of macrophages and T-cells, and profoundly reduced the TNF-α and other signaling pathways, including the NF-κB and MAP kinase pathways [43]. No hemorrhagic events were observed with the administration of GAGs. This report suggests that GAGs produce anti-inflammatory and immune response effects in a rat model of colitis (Figure 2 and Figure 3). Further analysis of these compounds for their utility in colon cancer prevention and treatment is warranted.

The dermatan sulfates from *S. plicata* (sulfated at carbon 4) and *P. nigra* (sulfated at carbon 6), which contain the same disaccharide core structure [α-l-IdoA(2SO_4_)-1→3β-d-GalNAc]*n*, are reported to be potent inhibitors of P-selectin [44,45]. In a thioglycollate-induced mouse model of peritonitis, dermatan sulfates showed drastic inhibition of metastasis of MC-38 colon carcinoma and B16-BL6 melanoma cells, regardless of the position of sulfation on the N-acetylgalactosamine, and also decreased the infiltration of inflammatory cells [44,45]. Fucosylated chondroitin sulfate (fCS) isolated from the sea cucumber *H. forskali* showed high affinity binding of the oversulfated CS/DS chains containing GlcAβ(1→3)GalNAcβ4,6S disaccharide units to L- and P-selectins. In addition, fCS isolated from this sea cucumber inhibited neutrophil migration [46].

## 3. Immunostimulatory and Anti-Tumor Functions of Sea Cucumber Extracts

A complex of monosulfated cucumariosides with cholesterol isolated from *Cucumaria japonica* demonstrated immunomodulatory properties in C57Bl6 mice (Figure 2). When administered in low doses, this complex displayed more than twofold stimulation of lysosomal activity on mouse macrophages (Figure 3). In addition, this complex significantly increased the animals’ resistance against bacterial infections elicited by *Y. pseudotuberculosis* or *S. aureus*. Furthermore, it increased phagocytosis and ROS formation, and stimulation of IL6 and TNF-α production was observed in lymphocytes [47].

Frondoside A is a monosulfated pentaoside, a component of the glycoside fraction isolated from *Cucumaria Frondosa*, and is reported to possess immunomodulatory properties when administered in subtoxic doses. A 0.2-μg dose of Frondoside A administered intra-peritoneally (i.p.) to Balb C mice increased lysosomal activity in macrophages in treated animals compared with vehicle-treated controls (Figure 3). Frondoside A increased cell-based immunity in these experiments; this immunity is an important feature for any preventive agent to improve the innate immune responses or in cases in which the host’s immune responses are hampered by cytotoxic agents against the antigen or tumor growth [48] (Figure 2). A triterpene glycoside isolated from *Cucumaria japonica*, cucumarioside A2-2, showed twofold increased stimulation of lysosomal activity and acted as a Ca^2+^ agonist in mouse macrophages [49] (Figure 3). Another report suggested similar increased immunostimulatory properties of triterpene glycosides from *C. japonica*, in which an increase in macrophage lysosomal activity was observed [50] (Figure 2 and Figure 3).

Triterpene glycosides isolated from sea cucumbers (*Mensamaria intercedens*) were evaluated for their anti-tumorigenic properties in a mouse model of S180 sarcoma and mouse Lewis lung cancer cell lines [51]. A hot water extract of sea cucumber (*Stichopus japonicus*) was reported to significantly inhibit proliferation and produce concentration-dependent cytotoxicity in human colon cancer Caco-2 cells [52]. Isolated sphingoid bases of sea cucumber (*Stichopus variegatus*) showed profound cytotoxic effects and decreased cell viability, and induced apoptosis via caspase 3 activity in DLD-1, WiDr, and Caco-2 human colon cancer cells (Figure 2). These *in vitro* studies of human colon cancer cells suggest that glycosides extracted from sea cucumbers may be good anti-tumor agents for the prevention and treatment of human colon cancer (Table 1).

**Table 1:** Sea cucumber extracts and their effects on various cancers in *in vitro* and *in vivo* models.

Compound	Sea cucumber	Effects	Type of cancer	Refs.
Triterpene glycosides	*Mensamaria intercedens*	Anti-tumorigenic	mouse model of S180 sarcoma and mouse Lewis lung cancer cell lines	[51]
Hot water extract	*Stichopus japonicas*	Anti-proliferation cytotoxic	Human colon cancer CaCo2 cells	[52]
Organic extracts	*Holothuria leucospilota*, *Holothuria scabra*, *Stichopus chloronotus*	Anti-proliferation	human A549 non-small lung cancer cells and C33A cervical cancer cells	[29]
sulfated triterpene glycosides	*Pearsonothuria graeffei*	Invasion, migration, decreased VEGF, MMP9, increased TIMP-1, decreased NF-κB	human hepatocellular liver carcinoma cells (HepG2) and human endothelial cells (ECV-304)	[53]
Frondoside A	*Cucumaria frondosa*	Anti-proliferation	Pancreatic cancer cells	[54]
Frondoside A	*Cucumaria frondosa*	Tumor inhibition, anti-proliferation, apoptosis, increased p21	Pancreatic cancer xenografts	[55]
Frondoside A + Gemcitabine	*Cucumaria frondosa*	Tumor inhibition, apoptosis, necrosis, Cas3,7 & 9 increase	Pancreatic cancer xenografts	[56]
Frondoside A	*Cucumaria frondosa*	Anti-proliferation, Cas3, 7 increase	Lung and breast cancer	[57]
Frondoside A	*Cucumaria frondosa*	Anti-angiogenesis, decreased CD31	Lung cancer xenografts	[58]
Frondoside A + Cisplatin	*Cucumaria frondosa*	Tumor inhibition	Lung cancer xenografts	[58]
Frondoside A	*Cucumaria frondosa*	Anti-proliferation Migration and invasion, increase in p53, Cas3/7	Breast cancer cells	[59]
Frondoside A	*Cucumaria frondosa*	Anti-tumor	Breast cancer xenografts	[59]
Frondoside A + Paclitaxel	*Cucumaria frondosa*	cytotoxic	Breast cancer cells	[59]
Frondoside A	*Cucumaria frondosa*	Anti-tumor and anti-metastatic, decrease ERK1/2	syngeneic murine model of metastatic breast cancer using Line 66.1	[60]
Polar fraction of Frondanol A5	*Cucumaria frondosa*	Anti-proliferation, inhibition of cell cycle, induce apoptosis	Pancreatic cancer cells	[61]
Frondanol A5	*Cucumaria frondosa*	Aberrant crypt inhibition, p21 in-crease, DNA fragmentation, apoptosis	AOM-induced rat colon cancer model	[30]
Frondanol A5	*Cucumaria frondosa*	p21 increase, G2/Minhibition, apoptosis	Human colon cancer cells HCT116	[30]
Frondanol A5	*Cucumaria frondosa*	Inhibition of small intestinal and colon tumors, increase in GILT expression, macrophage phagocytosis	Apc^Min/+^ colon cancer model	[31]

Three novel oligoglycosides (okhotosides B1, B2, and B3) were isolated from sea cucumber (*Cucumaria okhotensis*). Researchers also tested frondoside A, cucumarioside A2-5, and koreoside A against HeLa cells. Oligoglycosides showed a moderate effect on HeLa cells, but frondoside A had a profound effect against both HeLa and THP-1 tumor cells [62]. Another study reported the use of aqueous and organic extracts of three sea cucumbers (*Holothuria leucospilota*, *Holothuria scabra*, and *Stichopus chloronotus*) on human A549 non-small lung cancer cells and C33A cervical cancer cells [29] (Table 1). Organic extracts of all three sea cucumbers exerted anti-proliferative effects on both cell lines (Figure 2). These effects may be due to the presence of flavonoids and phenols, which are known to have anti-oxidant properties [29].

Zhao *et al.* 2010 reported the anti-tumor and anti-metastatic effects of two sulfated triterpene glycosides, holothurin A (HA) and 24-dehydroechinoside A (DHEA), isolated from sea cucumber species (*Pearsonothuria graeffei*) [53]. These compounds were tested *in vitro* and *in vivo*. Metastasis inhibition was due to suppression of matrix metallo-proteinase-9 (MMP-9) and vascular endothelial growth factor (VEGF), and an increase in tissue inhibitor of metalloproteinase-1 (TIMP-1) expression in treated samples compared with untreated samples. Both sulfated triterpene glycosides significantly inhibited adhesion and invasion of human hepatocellular liver carcinoma cells (HepG2) and human endothelial cells (ECV-304) in cell migration and invasion assays (Table 1). Furthermore, these compounds were observed to inhibit NF-κB expression. Hence, these sulfated triterpene glycosides have the potential for further development as anti-cancer therapies.

Frondoside A, (triterpenoid glycoside) isolated from *C. frondosa*, is reported to have anti-proliferative effects on human pancreatic cancer cell lines, AsPC-1 and S2013. Frondoside A was initially tested in a xenograft model using AsPC-1 pancreatic cancer cells. Significant tumor growth inhibition of AsPC-1 tumors was observed in athymic mice treated with frondoside A (10 μg/kg/day). In this study, Frondoside A inhibited proliferation and induced marked apoptosis and p21 expression, with increases in caspase 3, 7, and 9 [54] (Table 1). Furthermore, this agent was tested alone and in combination with gemcitabine *in vitro*, in which combination doses had greater effects than individual doses. These drug combinations were then tested in a xenograft mouse model of pancreatic cancer. AsPC-1 and S2013 human pancreatic cancer cells were allowed to form tumors in athymic mice. Post-tumor formation, these mice were treated with a combination of gemcitabine (4 mg/kg/dose) and frondoside A (100 μg/kg/day) for 30 days. A significant reduction in total tumor weight and tumor volume was observed in mice receiving the combination compared with those receiving placebo and single agent doses. Treated tumors showed extensive apoptosis and necrotic tissue, compared with untreated tumors (Table 1). These experiments suggested that frondoside A can be an effective adjuvant in therapy settings [55].

Frondoside A has also been shown to inhibit lung cancer (LNM35, A549, NCI-H460-Luc2 LNM35, A549, and Nand CI-H460-Luc2) and breast cancer (MDA-MB-435, MCF-7, and HepG2) cell proliferation when administered in doses of 0.01–5 µM. In addition, this compound increased cas 3/7 activities in LNM35 cells. Injecting Frondoside A (IP, 0.01 mg and 1 mg/kg) into athymic mice inoculated with LNM35 lung cancer cells resulted in tumor volumes reduced to 41% and 43% after 25 days of treatment. Furthermore, this agent showed great anti-angiogenic effects, in which it reduced CD31 staining in lung xenografts and in a chorioallantoic membrane (CAM) model. Significant inhibition of metastasis was observed with Frondoside A administration in a xenograft model and an *in vitro* matrigel invasion assay. Likewise, a combination of Frondoside A and cisplatin enhanced the therapeutic potential of cisplatin in an LNM35 tumor xenograft model [56] (Table 1). Hence, the sea cucumber extract Frondoside A may be useful in treating lung cancer patients.

Some studies have also shown that Frondoside A has good potential for breast cancer treatment. In *in vitro* studies using human estrogen receptor (ER)-negative MDA-MB-231 breast cancer cells, Frondoside A was observed to inhibit cell survival, migration, and invasion. *In vivo* studies using nude mice with MDA-MB-231 breast cancer tumors showed that Frondoside A can inhibit tumor growth. *In vitro* studies demonstrated that Frondoside A reduced cell viability and increased apoptosis via the Cas3/7 pathway, with a concentration-dependent increase in p53 (Table 1). In addition, Frondoside A reduced invasion of MDA-MB-231 tumor cells in a matrigel invasion assay.

This compound was also tested in an athymic mouse model using MDA-MB-231 tumor cells after the tumor reached a size of 200 mm^3^. Frondoside-A-treated animals showed a significant reduction in tumor volume of about 87%, compared with control animals. An *in vitro* analysis of Frondoside A (1 µM) in combination with Paclitaxel (32 nM) enhanced the cytotoxic effect of paclitaxel against ER-negative MDA-MB-231 breast cancer cells [57]. Xinrong Ma *et al.* reported the anti-metastatic potential of Frondoside A in a syngeneic murine model of metastatic breast cancer using Line 66.1, a highly tumorigenic and metastatic cell line isolated from Balb/cfC3H mouse [58]. These cells were injected into syngeneic Balb/cByJ mice. In this mouse model, Frondoside A was reported to be effective in reducing lung tumor metastases by 45% when mice were injected with the 66.1 cell line pretreated with Frondoside A (5 µM/L). Furthermore, 3H-PGE2 binding assays showed that Frondoside A antagonized PGE2 binding to both EP2 and EP4 receptors, with much higher affinity towards the EP4 receptor. A dose-dependent inhibition of cAMP formation was observed with Frondoside A use, thus demonstrating that this agent can block ERK1/2 activation induced by PGE2 [58]. This natural agent is an effective alternative to synthetic COX-2 inhibitors. Frondoside A is effective in lung cancer, pancreatic cancer, and breast cancer growth inhibition when used alone and in combination with other therapeutic agents. A polar fraction of Frondanol A5 was effective in inhibiting proliferation and cell cycle arrest in the G2/M phase and by inducing apoptosis in AsPC and S2013 pancreatic cancer cells [59] (Table 1). All of these findings suggest that Frondoside A isolated from *C. frondosa* is an effective nutraceutical that has potential as an anti-cancer agent.

We have been working with Frondanol A5, supplied and characterized by Coastside Bio Resources, isolated from sea cucumber, to evaluate its anti-tumorigenic potential in rodent models of colon carcinogenesis. Frondanol A5 is an isopropyl alcohol/water extract of the enzymatically hydrolyzed epithelia of the edible North Atlantic sea cucumber, *Cucumaria frondosa*, and is considered to be its most active principle component. Frondanol A5 contains several anti-cancer and anti-inflammatory agents, including monosulfated triterpenoid glycoside Frondoside A, disulfated glycoside Frondoside B, trisulfated glycoside Frondoside C, eicosapentaenoic acid, 12-methyltetradecanoic acid, and fucosylated chondroitin sulfate, as well as canthaxanthin/astaxanthin in small quantities. We have tested this agent in two doses (150 ppm and 450 ppm) in an AOM-induced rat model of colon cancer, with aberrant crypt foci (ACF) as an end point. This testing was conducted in parallel with a known anti-inflammatory agent, sulindac. We observed a significant inhibition of ACF/colon (34%–55%) and multi-crypt ACFs (48%–70%) with 150 ppm and 450 ppm, respectively. The efficacy of Frondanol A5 was superior to that of sulindac and was dose-dependent. Frondanol-A5-treated ACFs showed decreased PCNA and increased p21 expression and apoptosis, compared with untreated ACFs. In addition, H2AX phosphorylation, associated with DNA fragmentation and caspase 2 cleavage, was noted in treated human colon cancer HCT116 cells. Furthermore, this compound caused a G2-M arrest of HCT 166 cells with p21 upregulation [30] (Table 1). Thus, dietary administration of Frondanol A5 was shown to have chemopreventive effects in an AOM-induced rat model of colon cancer.

Further continuing our work with this agent, we tested Frondanol A5 in an APC^Min/+^ mouse model, which simulates human familial adenomatous polyposis, an inherited disorder that will result in the formation of multiple intestinal polyps. Dietary administration of Frondanol A5 (250 or 500 ppm) effectively decreased both small intestine and colon tumors (CTs) in APC^Min/+^ mice. We observed a dose-dependent decreased effect on small intestinal polyps (30%–50%), whereas we observed little difference in inhibition of colon tumors (65%–75%) with the two doses in APC^Min/+^ male mice. In female mice, both doses produced 80% inhibition of CTs. We reported an increase in immune responses in treated animals, in which the isolated macrophages showed increased phagocytosis and GILT expression, compared with untreated animals [31] (Table 1) (Figure 3). These data suggest that Frondanol A5 has the potential to improve innate immune responses during intestinal tumor formation and could be further developed for prevention and treatment.

Crude and purified extracts of sea cucumber *C. frondosa* have been studied for their effects on dendritic cell maturation. The fraction FCF1 contains four monosaccharide units: *N*-acetylneuraminic acid (NANA), mannose (Man), *N*-acetylglucosamine (GalNAc), and galactose (Gal). These were the main components, along with a minor amount of fucose (Fuc), found to be effective in maturing DCs *in vitro*, thus increasing Th17 signaling [60]. This study suggests the role of sulfated polysaccharide extracts in modulating innate immune responses. A detailed study regarding how this may influence the tumor microenvironment during tumor growth should be performed.

Another study suggested that an ethyl acetate solvent fraction of *Stichopus japonicus* (SCEA-F) possesses anti-inflammatory functions when tested in a Raw264.7 macrophage cell line. The results showed that SCEA-F reduced iNOS and COX-2, identified by reduced protein and RNA in treated cells. Furthermore, molecular pathway analysis revealed that the decrease in iNOS and COX-2 is due to inhibition of the MAPK signaling pathway in murine macrophages [61] (Table 1). It would be interesting to further analyze the effects of this sea cucumber extract in tumor models to determine its anti-tumorigenic properties.

Eicosapentaenoic acid-enriched phospholipids (EPA-enriched PL) isolated from the sea cucumber *Cucumaria frondosa* are reported to protect PC12 cells from oxidative stress [63]. The tumor microenvironment plus stromal components drive oxidative stress to stimulate tumor progression. Thus, bioactive components of sea cucumber extracts could be applied to repair oxidative damage during tumor formation. Most esterified phospholipids (PLs) in sea cucumber extracts are present as polyunsaturated fatty acids (PUFAs). Some researchers have shown that these PUFAs are highly absorbed by the intestine and have an improved capacity to be incorporated into cell membranes [64,65]. Phosphatidyl choline, a form of PL, is reported to possess significant anti-diabetic properties; decreased blood glucose and increased insulin secretion and glycogen synthesis was observed in diabetic rats [66]. As diabetes is a condition that will increase the risk of cancer, these types of bioactive components displaying anti-hyperglycemic properties could be applied or tested in tumor models for anti-tumor activities.

## 4. Conclusions

The isolation and use of bioactive compounds from sea cucumber extracts has received increasing attention for their potential anti-inflammatory and anti-tumorigenic properties and the possibility of applications to the treatment and prevention of human diseases, including cancers. These compounds are appealing because of their natural origin, long history of use as food, and lack of toxic effects. Although sea cucumber extracts are used for food and medicinal purposes, their exact functions have not yet been studied in detail. There is a need to develop methods to isolate and purify individual compounds and study their medicinal values, which could be applied to the pharmaceutical, cosmetic, and nutraceutical industries.

These compounds must be explored in appropriate animal models to provide empirical evidence of their functionality. The available data suggest the sea cucumber as a rich source of bioactive compounds, which makes it an ideal source for drug discovery, although much future analysis is necessary. Most isolated agents have been tested in small amounts and in combination with various components. Still, there is the potential to identify novel individual anti-cancer agents from various species of sea cucumbers.

Sea cucumber extracts have a promising future to be developed as functional foods. These extracts could be potential candidates for the prevention and treatment of cancers. The compositions and doses of sea cucumber extracts must be standardized for human clinical use, and individual agent analysis must be performed to determine the potential benefits for the prevention and treatment of inflammatory diseases and cancer.
